# “I have to be a man for my son”: The narrative uses of fatherhood in prison

**DOI:** 10.1177/14624745211018760

**Published:** 2021-05-21

**Authors:** William J Schultz, Sandra M Bucerius, Kevin D Haggerty

**Affiliations:** University of Alberta, Canada

**Keywords:** fatherhood, narrative criminology, prison, prison code, redemption

## Abstract

Research on incarcerated fathers tends to accentuate the harmful familial consequences of parental incarceration and discuss how having children might prompt incarcerated fathers to desist from crime. Less attention has focused on how narratives of fatherhood shape the day-to-day dynamics of incarceration. Drawing on 93 qualitative interviews with incarcerated fathers in Western Canada, we focus specifically on our participants’ parenting narratives. Such narratives are significant interventions in the world, allowing incarcerated fathers to frame their identities in particular ways while simultaneously shaping personal behaviour. Our research, 1. Identifies important fatherhood narratives provided by our participants, and 2. Details how such narratives operate in prison, allowing our participants to advance personal agendas that are themselves related to the dynamics of incarceration. In doing so, we provide insights into incarcerated fathers’ situations and advance criminological efforts to appreciate how different actors entangled in the criminal justice system conceive, manage, and narrate their situation.

## Introduction

Titus was large, heavily tattooed, and physically intimidating. A powerful man in his early forties, he was charismatic and had a well-documented talent for fighting, which he had parlayed into a high-ranking position in a local street gang. The surrounding halogen-lit concrete walls gave credence to Titus’s tales of drug deals and robbery: we spoke deep in the heart of one of Canada’s most volatile maximum-security prisons. Most of our conversation focused on Titus’s criminal career:

I hustle. I make money. That’s what I do. It’s not about dope or nothing, about girls or nothing. It’s just about I’ll go out and I’ll make $10,000 in a day. And I’ll give it to you because I’ll make $10,000 tomorrow. It don’t mean shit to me.

Titus’s reputation gave him considerable standing on the street and in prison, which he reflected in his biography. In fact, he intertwined his gang membership and identity so closely that we often paused to clarify whether the “brothers” Titus was describing were other gang members, or his biologically-related “brother-brothers,” as he would call them. Given the central role that Titus’s criminal activities played in his biography, the following transition in his narrative was a jarring surprise:

I was sitting in the penitentiary and my son came. He came to visit me, and he had this little [gang] tattoo, little eagle head. And he was like, “Dad just like you, warrior.” And my whole mind just went blank, and I was like: “Fuck that.” I don’t want this for my babies. I don’t want this life for my kids. I’m doing enough time for everybody. They don’t deserve to have this life. He grew up with a mom that was a prostitute. And she just hung herself a month and a half ago. And they’ve got a dad that’s looking at another ten years [in prison] that they’ve been waiting for thirteen years to get out.

Until this point in our discussion, Titus had exclusively framed himself as a tough and dangerous man on the streets, recounting his drug dealing accomplishments and penchant for violence. However, when he began discussing his children, his story’s narrative arc dramatically shifted, and he began foregrounding a different set of characterizations focused on himself as a parent. In particular, he began weaving a far more complex narrative of failure, loss, and redemption, one where his paternal identity provided him with the strength he needed to survive incarceration.

Children and family are a frequent topic of conversation for incarcerated people, and many researchers have commented on the pain incarcerated persons experience from family separation (Haney, 2018; Turney and Wildeman, 2013; Ugelvik, 2014). The academic literature on this topic emphasizes both the familial consequences of parental incarceration and the role fatherhood might play in encouraging men to desist from crime (McKay et al., 2019). Much of the research in this area approaches paternal incarceration as a moment where father/child bonds are irreparably damaged, sometimes with life-long consequences (Haney, 2018; Nurse, 2004). Alternatively, researchers portray prison as a potential “turning point” for incarcerated fathers, which may prompt a move away from crime-involved lifestyles (Edin and Nelson, 2013).

We approach the dynamics of incarcerated fatherhood from a different angle. Drawing on 93 qualitative interviews with incarcerated fathers in Western Canada, we focus on how men recount parenting narratives to deal with the stressful and painful experience of incarceration (Ugelvik, 2014). We consider such narratives significant interventions in the world, allowing individuals to frame themselves in important ways while simultaneously shaping subjectivities and personal behaviour. Consequently, our research aims to: 1. Identify the main fatherhood narratives provided by our incarcerated participants, and 2. Detail how such narratives operate inside prison, allowing our participants to advance personal agendas that are themselves related to the dynamics of incarceration. In doing so, we offer insights into imprisoned fathers’ situations, and advance criminological efforts to appreciate how different actors enmeshed in the criminal justice system narrate their situation (Copes et al., 2015; Fleetwood, 2015; Presser and Sandberg, 2015).

## Literature review

Researchers have traditionally treated the accounts provided by incarcerated fathers about parenting as benchmarks. In-prison accounts are contrasted to the post-release reality of their parenting situations – which means that the “truth” of what incarcerated men *do* in the community is contrasted with the accuracy of what they *say*, especially in the case of parenting failures (Arditti et al., 2005; Easterling et al., 2019; Edin and Nelson, 2013; Sandberg et al., 2020). These dialogues play a significant role in shaping outcomes for incarcerated people, as observers use them to frame both stereotypes and interventions around how men engage or fail to engage with their families (Haney, 2018; McKay et al., 2019).

Narrative criminological research has departed from this analytical strategy in important ways, highlighting the need to rethink the nature and importance of the accounts provided by offenders, victims, and criminal justice operatives (Presser, 2016). Such an approach draws attention to the personal and social significance of narratives, stressing that individuals interpret their experiences through accounts that are themselves related to people’s social positions (Warr, 2020). Narratives are therefore a form of social action, helping to constitute the reality they simultaneously describe. Importantly, narrative analyses bracket off the veracity of accounts, as “no matter what kind of stories are told, or whether they are true or false, they tell us something important about values, identities, cultures, [and] communities” (Sandberg, 2010: 455).

There are many ways to appreciate narratives relating to crime, deviance, and criminal justice. Such narratives can provide insights into the subculture of criminalized populations, revealing key normative expectations, cultural distinctions, and boundary work (Lamont and Molnár, 2002). On a personal level, narratives help fashion a sense of self-identity and subjectivity, both through publicly articulating a narrative for different audiences, and through crafting an internal self-narrative relating to one’s biography, motivations, and aspirations (McAdams and McLean, 2013). Narratives can also help instigate, sustain, or deter criminal behaviour. Maruna (2001), for example, suggests that such accounts play a prominent role in fostering personal transformation, although it is difficult to ascertain how consequential such narratives are. Likewise, Ward and Marshall (2007) have observed that adaptive narrative identities can aid in rehabilitation efforts.

As it pertains to fatherhood, urban ethnographers have detailed narratives of love and devotion that criminalized men provide about their children. These men consistently articulate narratives about their families, especially when discussing desistance and future plans (Bourgois, 2003; Bucerius, 2014; Contreras, 2013). For instance, Grundetjern et al. (2019) studied the fatherhood narratives of marginalized drug dealers in Norway. They found that most participants describe working hard to maintain paternal relationships with their children, despite drug use, criminalization, and a widespread failure to meet socially-conditioned fatherhood expectations. While such narratives may have a performative element (Goffman, 1959), such performances often serve to fashion an identity that does not revolve around criminal activities, and fosters the prospect of eventual desistence (Bucerius, 2014).

Individual perceptions and experiences typically condition narratives. However, new research has also demonstrated that power structures influence the content and direction of narratives. This is especially noticeable in prisons, where institutional observers view narratives – especially those connected to a rehabilitated identity – as a crucial indicator of penance and reform (Warr, 2020; Zhang and Dong, 2019). Consequently, prison officials sometimes view incarcerated individuals who do not articulate a repentant narrative as having failed to rehabilitate, meaning that personal narratives can directly influence release and parole decisions (Crewe et al., 2017). Research also demonstrates that while it is crucial to perform such narratives in specific institutional settings, authorities rarely test the sincerity of such accounts. Warr (2020) even suggests that incarcerated people may not even be able to explicitly state how and why they choose to present specific narratives, even when they can articulate the adverse consequences of refusing to perform the contextually appropriate narrative (Crewe et al., 2017; Wright et al., 2017).

Family status has a close relationship with this form of narrative work. Drawing on research conducted with incarcerated ethnic minority persons in a Norwegian remand facility, Ugelvik (2014) argues that incarceration creates a type of masculinity crisis, as men are prevented from performing common, socially-acceptable, and taken-for-granted markers of normative masculinity relating to parenting and providing for one’s family. Consequently, many incarcerated fathers experience identity crises, which prison officials use to pressure them to accede to the values of the wider Norwegian society (see also Easterling et al., 2019). However, Ugelvik discovered that incarcerated fathers resist these conforming pressures by crafting oppositional narratives that reclaim agency and masculine paternal identities. Despite being unable to play an active role in their children’s lives, men told stories about their families to reframe themselves as “strong, active, and responsible fathers who love their children more than anything and are willing to do everything for their sake” (Ugelvik, 2014: 165). This, in turn, helps men resist the coercive nature of institutionally-prescribed identities, while simultaneously addressing the crisis of masculinity engendered by incarceration.

In a similar vein, Sandberg et al. (2020) demonstrate that incarcerated men in Mexico narrated extensive stories of being a “good father,” which enable them to hold onto a prized and socially-acceptable identity despite their imprisonment. They suggest that narratives of parenthood represent a normalcy project of sorts, allowing incarcerated people to engage in pro-social identity work by referencing their status as loving and caring parents (Frederick, 2017). Narrating stories of fatherhood allowed men to reclaim their masculinity, as “being a good father is a crucial part of being a good man” (Sandberg et al., 2020: 2; see also Connell, 1995). Importantly, these narratives also allow incarcerated parents to look beyond the harshness of their current incarceration toward a nebulous-but-hopeful personal future (Easterling et al., 2019; Sandberg et al., 2020).

Although this research is a useful starting-point, work on fatherhood narratives among incarcerated men remains limited in scope. Specifically, existing research generally focuses on how narratives move incarcerated people’s focus *away* from prison, either voluntarily or due to the pressures presented by prison authorities (Sandberg et al., 2020; Warr, 2020). Missing from such accounts is how parenting narratives shape the day-to-day experience of serving time, and how fatherhood accounts relate to the social and institutional dynamics of prison life. Our analysis provides insight into these processes.

## Methods, sample, and analysis

We draw our data from semi-structured interviews with 93 incarcerated men, held in four provincial prisons in Western Canada^1^. These interviews are part of a larger project that interviewed a sample of 495 men and 92 women in provincial prisons^2^. In Canada, these institutions detain legally innocent people who are awaiting trial in custody rather than in the community. Such individuals are referred to as “remand prisoners,” and they comprise a diverse population of people who have committed minor offences, such as having failed to pay outstanding fines, as well as those who have committed serious crimes, such as murder. Provincial prisons also detain individuals sentenced to a term of incarceration of up to two years. We recruited our interview participants from both remand and sentenced facilities.

To recruit participants, we made announcements on the individual living units of each prison. Nearly everyone incarcerated on the units signed up, either out of intrinsic interest or because the study offered “something to do” in institutions with little programming and few recreational opportunities. We conducted semi-structured interviews in private rooms on or near the living units. Using a generalized interview guide, we asked participants about their life history, prison culture, and relationship with family and loved ones, among other topics.

The 93 participants whose accounts inform this article spoke extensively about fatherhood in their interviews, providing deep and rich data about their paternal identity. They ranged from an 18-year-old with one child, to men in their 50s and 60s who discussed dozens of children and grandchildren. Thirty-five of these 93 participants (38%) self-identified as Indigenous, while 29 (or 31%) identified as Caucasian. Ten identified as Black, Asian, or “Middle Eastern,” while 19 did not specify their ethnic background. These profiles are representative of the massive over-incarceration of Indigenous men and women in Western Canada, where Indigenous people consistently account for between 40 and 75% of the prison population despite comprising only 10% of the general populace (Malakieh, 2020).

In line with the interviewing practices standard in narrative criminology, we allowed individuals’ unique accounts to drive the discussion. We commenced each interview by inviting participants to “tell us whatever you would like us to know about yourself,” leaving it up to the participants what they wanted to share at the outset. As the discussion progressed, we prompted into topics relating to our participants’ upbringing, relationships, spouses, children and other support networks. This resulted in an interactional situation that enabled us to collect and co-create a larger story-arc of how fatherhood identities shaped their incarceration experiences (Presser, 2005; Presser and Sandberg, 2015; Sandberg et al., 2020).

After we completed the interviews, we assigned randomly-generated pseudonyms to each participant and transcribed the interviews verbatim. Following transcription, the authors and three research assistants reread each interview, identifying themes and narratives emerging from the data (Charmaz, 2014). We used these themes to create a coding scheme, which we tested, adjusted, and redefined against randomly-selected transcripts until we consistently reached between 85 and 90% inter-coder overlap. Once we consistently met this target, we used NVivo 11 software to thematically code each of the 93 interviews line-by-line. This detailed coding allowed us to identify three unique narrative themes around fatherhood, which we discuss below.

## Findings

Our participants articulated three fatherhood narratives relating to their prison experience. These were, 1. redemption, 2. illicit provider, and 3. departures from the prison code. Not all men identified in the same way with each narrative: some identified with more than one, while others emphasized a single, dominant narrative. However, the favourable cultural meanings of fatherhood provided opportunities for participants to invoke their paternal status to advance different personal goals (Sandberg et al., 2020; Warr, 2020). These narratives often included efforts to project a particular vision of masculinity, referencing such stereotypical paternal attributes as courage, financial support, and paternalistic love (Connell, 1995; Grundetjern et al., 2019). For our purposes, we do not treat masculinity as a distinct fatherhood narrative, but as a pervasive and almost ubiquitous aspect of prison life, inextricably linked with each of the three narratives we identify (Bartlett and Eriksson, 2019; Evans and Wallace, 2008).

### Redemption narratives

Facing the daily challenges of imprisonment, fathers in our sample narratively reframed their family experiences, using their paternal status to reclaim the stressful and often-humiliating experience of incarceration (Crewe, 2011; Sykes, 1958). In keeping with past research, incarcerated fathers detailed extensive stories of suffering and anomie arising from family disconnection (Merton, 1938; Sandberg et al., 2020; Sykes, 1958). However, in a unique twist, men also described fatherhood narratives as something that helped them meaningfully deal with the painful reality of serving time, especially when it came to managing the crisis of masculinity precipitated by incarceration (Ugelvik, 2014). Julian provided a clear example. He recounted his struggles with substance misuse, which culminated in three separate suicide attempts. Immediately after describing his most recent suicide attempt – which was part of the incident which had led to his incarceration – Julian gestured to the walls of the interview room and said that in prison,

I found myself again. Sometimes you have to be alone to find out who you really are. I found myself again. I found out who I really was, why I was struggling. Why was I always blaming myself for this and that? And I told myself, I never even learned how to not be in here. They just use me and throw me away. Right? I have to be a man for my son. That’s why I say jail saved me.

Men like Julian used their parental status to reframe incarceration, narrating why “this time” was different, and pointing to their fatherhood as motivating their quests for personal transformation. Dylan went even further:

I think everyone should come to jail once … I lost my [four] kids, and it was downhill from there right. But, I mean like jail, it’s almost like it helps me to get back to [being] me … once you get over the first week or two, it’s almost – for me I rebuild myself.

Men like Dylan and Julian characterized prison as an important and redemptive moment in their journeys as fathers: Dylan “rebuilt” himself in prison, while Julian “learned his lesson” about how to “be a man for his son.”

Children served as redemptive motifs within these narratives, and stood at the heart of the changes incarcerated fathers were striving towards. Stan, a 29-year-old father of two who had grown up in a violent organized crime family, put it this way:

I learned six different recipes to cook methamphetamines. I’ve never done it, but off the top of the head I can tell you recipes right now. I don’t spread that information anymore, because I’m on a different path. I’m here now to make a positive impact. I have two seven-year-old daughters. Even though my father is who he is, and I still can travel that path, I won’t. I made that decision a long time ago, but I was still stuck in between. Now, coming here, I’ve realized I’ve built the tools to enable myself to build a whole new ego. I’ve created a new identity to work with … I’ve made something new.

Narratives like Stan’s allowed men to draw a positive picture of their lives, their futures, and their present. Incarcerated fathers used these stories to portray themselves as changed or changing men, who were stepping away from destructive street lives. Crucially, these narratives allowed men to reclaim agency over their incarceration and prison experience, while simultaneously addressing the masculinity crises engendered by being forcibly removed from their lives and families (Connell, 1995; Sandberg et al., 2020). Rather than succumbing to the anomie of long-term prison sentences (Crewe, 2011; Wright et al., 2017), stories of redemption allowed fathers to identify some larger meaning or purpose in their punishment. Children stood at the center of these narratives, providing motivation and sustaining change. For instance, in the excerpt above, Stan drew a direct contrast between what he *could* do, and what he was *choosing* to do, placing his daughters at the heart of his redemptive narrative and emphasizing his agency in building a future and new identity for their benefit.

For many participants, fatherhood narratives were aspirational, demonstrating what men dreamed of and hoped for after release (Sandberg et al., 2020). Simultaneously, the narratives served as an important resource, allowing men to coherently articulate and manage their experiences in prison. Instead of allowing incarceration to define their existence, fathers narrated their desires for change and framed their prison-based efforts to reform or self-improve with reference to their children. Stan and Jared each described the changes they had made as evidence that “this time” they would “go straight” in the future, irrespective of structural and historical barriers (Haney, 2018). By doing so, they reimagined their incarceration, framing it as a purposeful and meaningful experience that allowed them to become better fathers and people.

### Illicit provision narratives

Incarcerated fathers did not universally embrace redemptive narratives, especially when discussing the prospect of leaving criminal lifestyles. A subset provided a counter-narrative, portraying criminal careers as an essential part of their paternal identity, even during incarceration. This “illicit provider” orientation was particularly apparent amongst men who took advantage of prison’s financial opportunities. Incarcerated men could make considerable sums of money through drug-dealing and other forms of hustling (Bourgois, 2003; Crewe, 2005; Sandberg, 2008). Marijuana, crystal methamphetamine, tobacco, and fentanyl were widely available, selling for approximately ten times their street value (Bucerius and Haggerty, 2019). Yet, the narratives of the fathers involved in this trade did not center on money. Instead, men like Stephan rationalized such illicit commerce by accentuating their parenting responsibilities: “The only reason why I sell drugs, it’s sad to say, I got family, I got kids. I got two kids, a daughter, and a son. And fuck, shit’s expensive.”

Stephan’s account was typical of the illicit provider fatherhood narrative. Men articulating this narrative portrayed criminal activity as a means of fulfilling the socially-acceptable role of father as the provider for the family (Grundetjern et al., 2019; Sandberg et al., 2020). These men made no apology for dealing drugs: instead, they described how in-prison dealing allowed them to fulfil their familial duties. In Etienne’s words:

Etienne: I’ve been in for seven months, right? So, I’m not making no money really and so [drug dealing’s] my bread and butter [ … ] I’ve kids to take care of on the street, right?

I: Okay. So the [drug] money that is made in here is used to also help family?

Etienne: That’s all I do it for. Otherwise, I wouldn’t do it. I really wouldn’t care. I would just eat my three meals a day and do my little bit of gambling or whatever and that’s it. But I’ve got a daughter and a son, right?

In this narrative, incarcerated fathers characterized their children as a motivating force behind why they took the risks of drug dealing. The larger prison population was aware of this rationale and viewed the “illicit provider” narrative skeptically. Yet, dealers like Etienne insisted on the connection between fatherhood and drug profits. Like Etienne, men who voiced this narrative portrayed drug dealing as something they *regretfully* engaged in – compelled by the demands of parenting, and the honorable societal expectation that a father should provide for his children (Crewe, 2005; Grundetjern et al., 2019). This narrative helped create a masculinized, quasi-heroic self-portrait. Fathers framed themselves as altruistically assuming significant personal risks in dealing drugs – ultimately doing so to benefit their children and families (Connell, 1995).

The “illicit provider” narrative also contained subtle nuances that drew upon and reinforced themes of masculine self-sufficiency and good citizenship. As Tyler put it:

I dealt hashish in [the penitentiary] for four years. Regularly I was grossing $12,000 a month profit, right? Wife, two little kids, right? You know, I didn’t rely on the welfare system to take care of our family, my family.

Here, Tyler’s reference to welfare and state support is an interesting gloss on the masculine “provider” narrative. Like the taint of being a “deadbeat dad” (Grundetjern et al., 2019), for some incarcerated fathers the prospect that their family was (or might be) on welfare represented a stigmatizing personal failure, negatively shaping their paternal identity (Cammett, 2014; Hansen et al., 2014; Miller, 2010). As Lachlan put it:

I was also an institutional drug dealer. I provided the drug subculture with just hashish, right, I didn’t dissipate hard drugs or any of that, but I needed to support my young girlfriend an’ my daughter … we chose not to burden society with what I did, more financially speaking, right – like, it was somewhat moderately lucrative.

Lachlan used the “no-welfare” gloss as part of a larger claim to a moral high ground: drug trafficking was only “moderately lucrative” and allowed him “not to burden society.” He never “dissipated hard drugs,” only hashish that an impersonal “prison subculture” demanded. Through careful narrative framing, Lachlan places himself in the ranks of the “morally conscious proper criminals” who only do “good” crimes (Ugelvik, 2015: 23), while simultaneously framing himself as a caring father whose actions were based in concepts of honor, moral rectitude, and familial provision (Bucerius, 2007). These narratives allowed Lachlan and Tyler to reclaim positive identity markers stripped away through the process of incarceration. By reproducing societal narratives about fathers as providers, both men re-established their status as “good” citizens, fathers, and men, thereby repudiating cultural stereotypes that portray incarcerated men as morally-suspect deadbeats (Sandberg et al., 2020).

### Departures from the prison code

Prisonization – individual socialization into a counter-cultural identity, shaping both in-prison experiences and post-prison rehabilitative efforts – has stood at the center of carceral research for over 80 years (Clemmer, 1940; Martin, 2018; Shlosberg et al., 2018). When examining how people “become” a prisoner, criminologists have focused on the “convict code” (also known as the “prison code” or “inmate code”), which regulates interpersonal and organizational dynamics among incarcerated persons (Clemmer, 1940; Irwin and Cressey, 1962; Mitchell et al., 2017; Sykes and Messinger, 1960). Researchers have identified five consistent themes in the prison code (Crewe, 2013; Higgs, 2014): a prisoner should 1. never “rat” (inform/snitch) on another inmate; 2. hold anti-authority views and avoid prison staff; 3. be loyal to one another (sometimes called being “solid”); 4. be tough and display manliness; and 5. do their own time (mind their own business). The prison code prospectively shapes behaviour, while also serving as a resource that allows for after-the-fact rationalizations and explanations of behaviour (Jimerson and Oware, 2006). It plays a significant role in shaping prison hierarchies and prompting or justifying violence within prisons (Skarbek, 2014; Trammell, 2012).

Failure to follow subcultural “rules” carried distinct and immediate consequences for our participants. Men who violated “the code” risked being assaulted, referred to and treated as the “unit bitch,” or forced to accept the humiliating status of “PC,” or protective custody, for their own safety (see Copes et al., 2013; Garot, 2009; Scott and Lyman, 1968). As with any set of rules, it was possible to resist some aspects of the prison code, but doing so could be risky and required considerable subcultural acumen. Incarcerated men had to narrate either resistance or creative interpretations, both to themselves and to other inmates, or face consequences for failing to conform (Ugelvik, 2016). In this context, narratives like James’s were unexpected and intriguing:

I promised my son I wouldn’t [return to prison again], so I can’t. He’s six years old. He told me he doesn’t want me here no more, so I promised I’d never come back … That’s why I don’t fight no more. I’ve got to keep my cool in here now … Last week buddy came and called me a goof, squid, everything to my face, and he’s pushing me [to fight]. I said, “Buddy, you’re not worth it. Fuck off and get away from me.” … It doesn’t matter. They can hit me. I don’t care. I’m not going to swing back, unless they get too much. Then I’m going to protect myself. Right now, it’s not worth it. I’m going home to my kid, and there’s no ifs, ands, or buts about that.

James’s story was memorable. He was in his early 30s at the time of our interview, and had an extensive criminal history, including time in juvenile detention for killing his father: “I’ve done my whole life [in prison]. The longest I’ve been out on the street is 96 days … From 12 years old I did five years [ … ] because I killed my father.” Respected and feared by his peers, James had a tremendous amount of street capital and was regularly engaged in confrontations (Sandberg, 2008).

Yet, James was attempting to change his identity and leave his history behind him. Narratives about fatherhood helped him to articulate this transformation. Identifying with a narrative about being a caring father who kept his promises to his child allowed James to rationalize acting in ways that rejected his priority identity, such as walking away when insulted, instead of fighting. This was no mean feat: “goof” was the most incendiary label in the prisons we studied, and represented a direct challenge to one’s manhood. Failing to respond was perceived as cowardice; failing to respond in front of a large group of witnesses – and the scenario James described happened during a crowded poker game on a maximum-security unit – usually led to victimization and violence (Trammell, 2012).

Yet, James presented a narrative that emphasized his loyalty to his son rather than to the code, which allowed him to rationalize departing from standard masculine subcultural expectations. The strength of this narrative, and its influence in the context of the prison culture, was demonstrated by how James’ peers responded to his statement. According to him, the other men on his unit were aware of his promise to his son, so when he was challenged

The whole poker table stood up and told him, ‘Listen, he said to fuck off. Now fuck off, or we’re all going to jump you.’ That was respect right there. For the whole table to step up like that is pretty good.

This supportive response speaks to the strength of the fatherhood narratives James employed: not only did his stance allow him to avoid violence, but his commitment to doing the “right thing” for his child – *especially* in contrast to his typical, violent response to such challenges – earned him respect from his peers, more than he would have gained by fighting.

James’ situation was extraordinary but not unusual. Men positioned fatherhood narratives as a higher calling – something so strong that they had no choice except to break code-based behavioural expectations. Again, there could be a type of heroism motif embedded in these accounts, with fathers accentuating the considerable risks they assumed to advance their children’s interests. Titus, who we quoted in the introduction about leaving a gang for the benefit of his children, gives a sense of this by describing the potential consequences of that decision:

When I seen that [my son’s gang tattoo] I went back to them [my gang], and I was like, ‘I’m done. You guys do whatever you guys want to do. Fuckin’ stab me, do whatever you’ve got to do. I’m walking out this door.’

Likewise, Alfred described how he used his status as a father to exit a violent white supremacist group:

I left the Aryans. I’m on my own now. I’m independent … A couple of them don’t like the fact that I’m talking to other cultures, but I’m not doing it for them. I’m doing it because I have kids at home.

For fathers like Alfred and Titus, their accounts of embracing fatherhood allowed them to live up to their own and broader societal expectations of normative parenting. It supported their motivation to leave violent groups and provided a vocabulary that helped to ease and justify such a transformation, even when the consequences of such an action could be severe (Skarbek, 2014).

Our participants used fatherhood narratives as a resource to help them justify violating expectations of the code they might otherwise embrace, and even reinforce (McAdams and McLean, 2013). One of the more extreme examples of this process related to the “don’t snitch” maxim, which serves as a golden rule for criminally-involved populations (Pyrooz et al., 2021). Receiving the label of “rat” or “snitch” can be akin to a death sentence in some settings, including prison (Seabrook and Stewart, 2014). Unsurprisingly, few of our participants discussed how they personally engaged in snitching behaviour. But, the ones who did directly connected their normative breach to fatherhood. In one notable instance, a middle-aged man described how he was becoming a federal informant and would provide information to the Royal Canadian Mounted Police (RCMP) about a violent international criminal organization in exchange for a reduced sentence. While he discussed the risks involved and was uncomfortable with violating subcultural prohibitions on snitching, he explained at great length that he was otherwise facing a 20-year sentence and could not handle the prospect of being apart from his children for that long.

## Discussion and conclusion

Criminologists have dedicated considerable attention to studying incarcerated parents. The scholarship on incarcerated fathers has foregrounded the social, relational, and economic challenges that incarceration poses for fathers and families (Grundetjern et al., 2019; McKay et al., 2019; Sandberg et al., 2020; Turner, 2017). However, fatherhood is not merely a set of biological, financial, or familial relationships; it is also a symbolically rich social identity, performed and made understandable through personal narratives, and deeply intertwined with masculinity (Connell, 1995). Like many disadvantaged men, our participants used their status as fathers to align themselves with a respected and socially-normative identity – one which, unlike many positive symbols, was comparatively accessible to them (Bourgois, 2003; Contreras, 2013; Edin and Nelson, 2013; Grundetjern et al., 2019).

Becoming a father was a pivotal moment in our participants’ life-course narratives. Family, and especially children, occupied a core place in how they conveyed their identity. Their status as fathers was a source of encouragement and possibilities, providing a valorized status and a meaningful identity (Edin and Nelson, 2013). With incarceration limiting their abilities to perform conventional fatherhood roles (Meek, 2011), such narratives assumed added significance as a means by which incarcerated fathers could project a masculine parental identity while physically separated from their families (Ugelvik, 2014).

Fatherhood narratives often had a redemptive structure, with men portraying incarceration as their biographical low point but projecting an ascent from their current station. Men used these accounts to fashion a “new” normative self that had undergone a positive personal transformation and presented a hopeful future (Sandberg et al., 2020). In such accounts, imprisonment – while involuntary and profoundly unwelcome – became one moment in a larger story of personal decline and perhaps failure, but also of ultimate recovery and vindication. Imprisoned men were able to describe changes they had made while detained, allowing them to present incarceration as a crucial step on their reformative journey to a new and better self. The broader psychological study of narratives has demonstrated that individuals who provide such “redemption tales” have a greater sense of psychological wellbeing, even if enacting such narratives is challenging (McAdams et al., 2001).

The narrative uses of fatherhood were highly pliable in prison. So, while some incarcerated fathers referenced their children to justify disengaging from harmful and criminal behaviours, others invoked children to rationalize their ongoing illegal actions as counter-cultural means to achieve stereotypical pro-social ends (Bourgois, 2003). In some ways, this strategic and contextual use of narratives to support individual ambitions resembles a lay or simplified version of Merton’s (1938) theory of anomie, specifically his notion of innovation, where individuals engage in illegitimate activities to fulfil socially desirable ends. This is a common trope amongst criminalized populations, with many ethnographers having demonstrated that drug dealers, for example, frequently reference the “American Dream” when describing their motivations (Bourgois, 2003; Contreras, 2013; Sandberg, 2008) or justify their actions by pointing to others, who are – in their minds – involved in far worse crimes (Bucerius, 2014; Ugelvik, 2015). The fathers who aligned themselves with the “illicit provider” narrative did not present the money they earned from selling drugs as a distinctive goal, nor as a means towards conspicuous consumption. Instead, drug profits represented a way to provide for their children. This allowed them to claim a positive parenting identity and neutralize social stigma attached to their drug dealing (Sykes and Matza, 1957). However, this narrative does more than simply negate stigma. The portrait of dealers as “good providers” instills their actions with a commendable, even heroic, quality, as it implies they are assuming considerable personal and legal risk to support their children.

Crucially, these narratives motivate action, supporting and advancing the possibilities of change (Maruna, 2001). The scope for such action is undoubtedly circumscribed given the restrictions of prison. Still, incarcerated fathers referenced their fatherhood commitments as motivating them to do such things as to leave gangs, avoid fights, and even break the foundational “no-snitching” rule. The snitching dialogue, in particular, presents an interesting tension point in relation to prison codes. Research suggests that the widespread solidarity against snitching in prison is, in part, a result of masculine expectations about being solid, tough, and doing the right thing, and consistently identifies the crucial role masculinity plays in shaping the larger prison code (Crewe, 2013; Ugelvik, 2014, 2016). Yet, our participants point to masculinized narratives around being strong, solid, and “doing the right thing” – which are usually invoked as expectations of the prison code – to justify and explain why they might back down from fights and breach the “no-snitching” rule.

The tension in these positions is intriguing: according to the masculinized prison code, the “right” thing to do is to defend your honor and fight when challenged. In contrast, as a father the “right” thing to do may be to ignore slights and provocations, or (in the extreme case we identified) even provide information to the police, to continue the transformation into becoming a better man for one’s children. Masculinity proves flexible here: “being a man” in prison may be interpreted in unexpected ways when masculine behavioural codes are narrated through the lens of fatherhood (Ugelvik, 2014). What is more, such narratives may be acceptable to incarcerated peers. Previous research has examined how criminally-involved men in the community imagine a future away from their criminal life-style by invoking narratives about family and children (Bucerius, 2014), and how gang members have referred to the higher calling of religion to move away from gang life (O’Neill, 2014). Our study adds a new dimension to such processes, with incarcerated men like James and Titus presenting fatherhood narratives as a higher calling in prison, providing an acceptable justification for opting out of certain expectations of the prison code. Of course, the significant degree of street capital James and Titus possessed likely played a key role in shaping how peers responded to their fatherhood narratives (Sandberg, 2008). As such, it remains an open question whether an unproven, newly-incarcerated person could effectively use fatherhood narratives to escape confrontation. However, for men who were well-established in the prison subculture, fatherhood represented an acceptable way to narrate personal and behavioural changes, allowing them to step outside of the otherwise accepted informal rules governing prison dynamics. By presenting their children as the rationale behind their actions, our participants justified atypical code-violating decisions to different audiences. As interviewers, we were undoubtedly one such audience, but they themselves were also simultaneously a vitally important audience for their portraits of an honourable self and hopeful future, something which justified present action.

The narratives about fatherhood in prison ultimately offer insights into the hopes and dreams of a large group of incarcerated men. Irrespective of whether these narratives point to a viable future (Sandberg, 2010), they offer an aspirational vision and sense of meaning for incarcerated and often vulnerable people. In some cases, these narratives even help fathers resist and ignore damaging subcultural expectations associated with incarceration (Crewe et al., 2017; Nurse, 2004; Wright et al., 2017). Overall, our research demonstrates that fatherhood narratives play a far larger role for incarcerated men than previously believed. As a result, fatherhood narratives may provide us with a new starting point to engage with incarcerated individuals, providing beneficial insights into how we understand incarceration experiences, the larger dynamics of masculinity in prison, and avenues toward and around desistence more generally.

## Notes

1 As part of our research agreement, we do not identify the province where we conducted our research.

2 The University of Alberta provided research ethics approval in 2016.
